# Supplementary material for: Temporal trends of SARS-CoV-2 seroprevalence during the first wave of the COVID-19 epidemic in Kenya
Source: Nat Commun. 2021 Jun 25;12:3966. doi: 10.1038/s41467-021-24062-3 (PMC8233334; doi:10.1038/s41467-021-24062-3)
Supplement: Supplementary file 1 [file 41467_2021_24062_MOESM2_ESM.pdf]

Supplementary Information

Title: Temporal trends of SARS-CoV-2 seroprevalence during the first wave of the COVID-19 epidemic in Kenya.

Authors: Ifedayo M.O. Adetifa[1,2*§], Sophie Uyoga[1*§], John Gitonga[1§], Daisy Mugo[1§], Mark Otiende[1], James Nyagwange[1], Henry K. Karanja[1], James Tuju[1], Perpetual Wanjiku[1], Rashid Aman[3], Mercy Mwangangi[3], Patrick Amoth[3], Kadondi Kasera[3], Wangari Ng'ang'a[4], Charles Rombo[5], Christine Yegon[5], Khamisi Kithi[5], Elizabeth Odhiambo[5], Thomas Rotich[5], Irene Orgut[5], Sammy Kihara[5], Christian Bottomley[2], Eunice W. Kagucia[1], Katherine E. Gallagher[1,2], Anthony Etyang[1], Shirine Voller[1,2], Teresa Lambe[6], Daniel Wright[6], Edwine Barasa[1], Benjamin Tsofa[1], Philip Bejon[1,6], Lynette I. Ochola-Oyier[1], Ambrose Agweyu[1†], J. Anthony G. Scott[1,2,6†], George M. Warimwe[1,6†]

Affiliations:

[1] KEMRI-Wellcome Trust Research Programme, Kilifi, Kenya.

[2] Department of Infectious Diseases Epidemiology, London School of Hygiene and Tropical Medicine, Keppel Street, London, United Kingdom.

[3] Ministry of Health, Government of Kenya, Nairobi, Kenya.

[4] Presidential Policy & Strategy Unit, The Presidency, Government of Kenya.

[5] Kenya National Blood Transfusion Services, Ministry of Health, Nairobi, Kenya.

[6] Nuffield Department of Medicine, Oxford University, United Kingdom.

*Correspondence to: IAdetifa@kemri-wellcome.org and suyoga@kemri-wellcome.org

Supplementary Figure 1. Participants flow diagram for SARS-CoV-2 seroprevalence study of blood donors in Kenya.

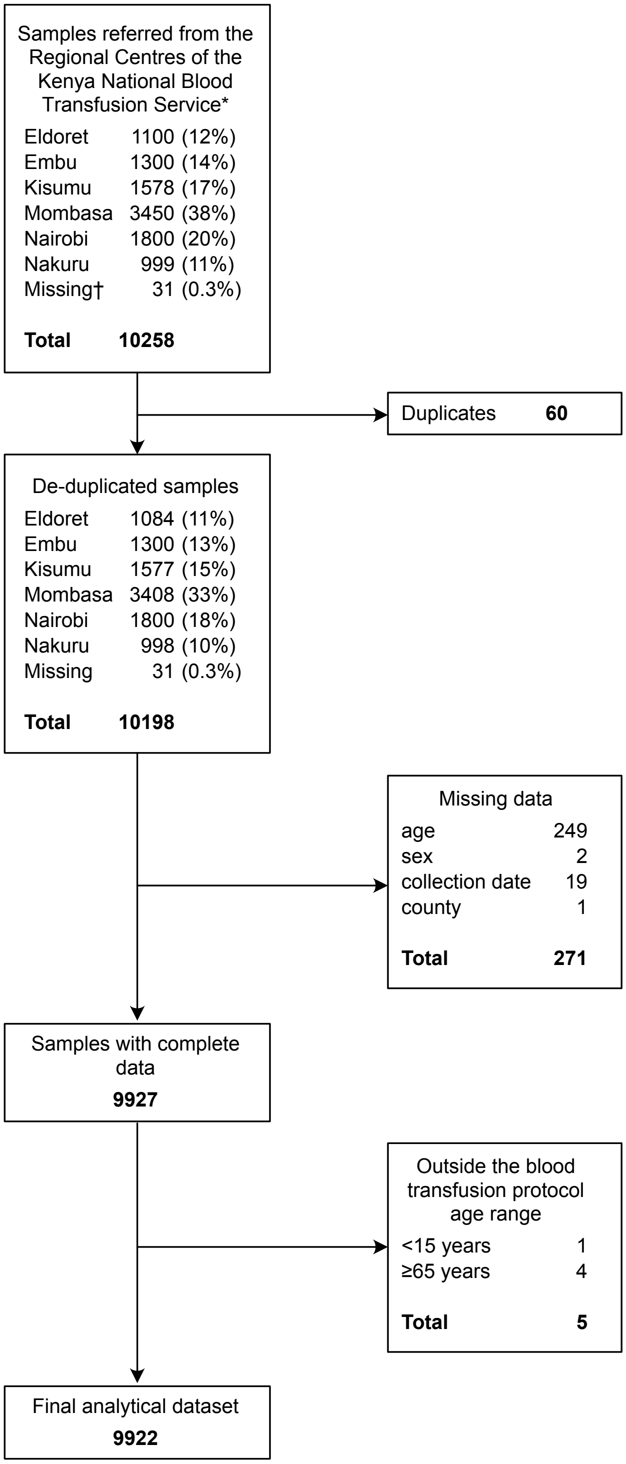

*3174 samples reported in an earlier publication are included in this analysis[1]
†All 31 records with missing information on Regional Centre were excluded further down the algorithm because they all lacked information on donor age.

Supplementary Figure 2 Frequency of samples collected across the study period by region.

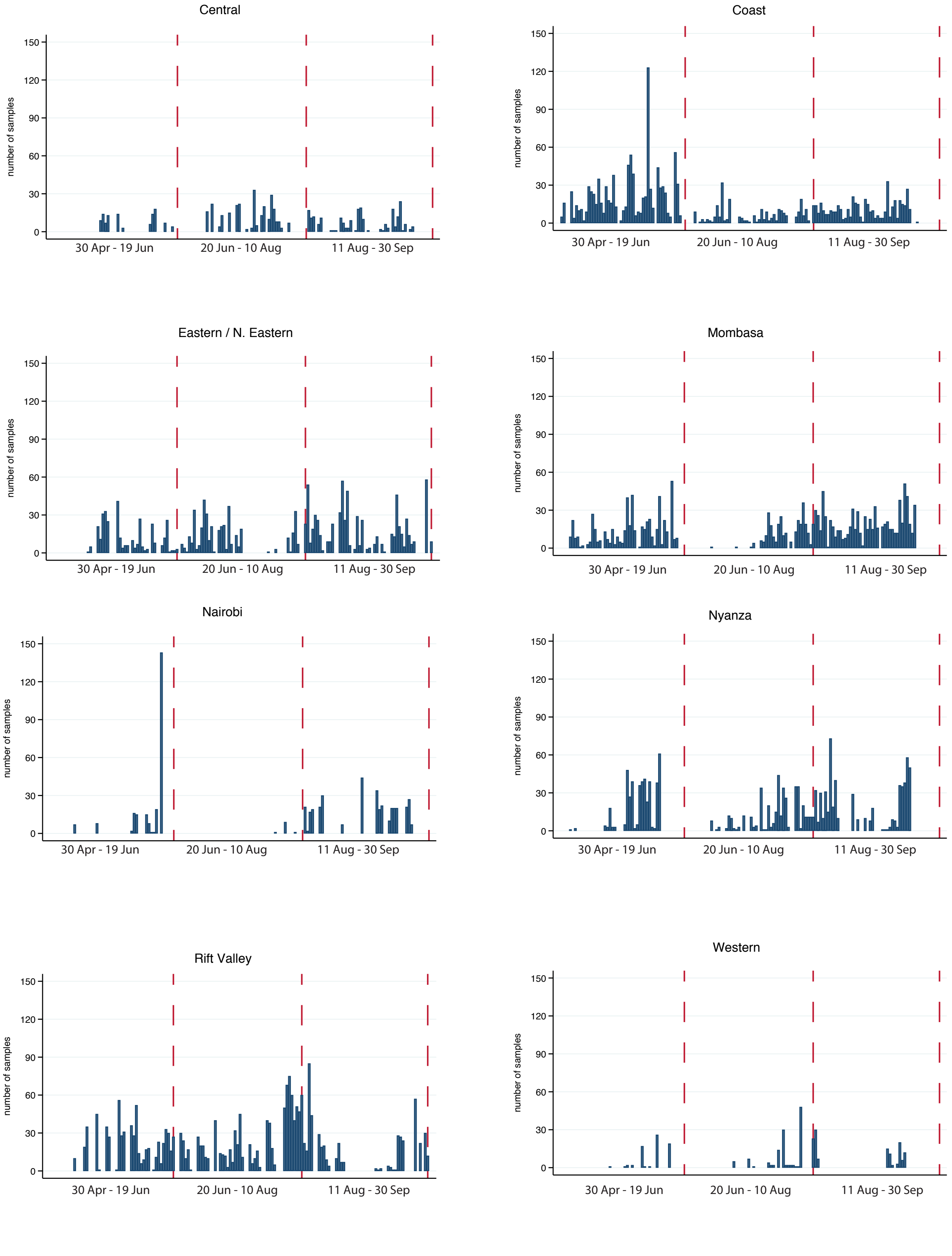

Supplementary Table 1. General characteristics of the study population compared to the national population of Kenya.

Demographic variables		Blood transfusion samples		Kenya National Census 2019	
		N	%	N	%
Age	15-24 years	2,763	27.9	9,733,174	35.8
	25-34 years	3,902	39.3	7,424,967	27.3
	35-44 years	2,261	22.8	4,909,191	18.1
	45-54 years	794	8.0	3,094,771	11.4
	55-64 years	202	2.0	1,988,062	7.3
Sex	Male	8,019	80.8	13,388,243	49.3
	Female	1,903	19.2	13,761,922	50.7
Region	Central	606	6.1	3,452,213	12.7
	Coast	1,680	16.9	5,176,080	19.1
	Eastern / N. Eastern	1,482	14.9	792,072	2.9
	Mombasa	1,654	16.7	1,671,097	6.2
	Nairobi	607	6.1	3,002,314	11.1
	Nyanza	1,433	14.4	3,363,813	12.4
	Rift Valley	2,138	21.6	7,035,581	25.9
	Western	322	3.3	2,656,995	9.8
	National	9,922		27,150,165	

N is the number of individuals in each stratum. % are column percentages

Supplementary Table 2. A comparison of the general characteristics and SARS-CoV-2 seroprevalence in the blood donor populations in Kenya

	Family Replacement Donors				Voluntary Non-Remunerated Donors			
	Number sampled	% of sample	Antibody positive	Seroprevalence (%)	Number sampled	% of sample	Antibody positive	Seroprevalence (%)
All donors	9,632	100	899	9.3	290	100	29	10.0
Male	7,780	80.8	741	9.5	239	82.4	21	8.8
Female	1,852	19.2	158	8.5	51	17.6	8	15.7
15-24 years	2,693	28.0	232	8.6	70	24.1	9	12.9
25-34 years	3,793	39.4	368	9.7	109	37.6	11	10.1
35-44 years	2,191	22.7	219	10.0	70	24.1	5	7.1
45-54 years	765	7.9	64	8.4	29	10.0	2	6.9
55-64 years	190	2.0	16	8.4	12	4.2	2	16.7

Voluntary Non-Remunerated Donors (VNRDs), who donate blood at community-based ‘blood drives’ comprised only 3% (290/9922) of our sample of donors during the pandemic; almost all donors in 2020 were Family Replacement Donors (FRDs) who provide a unit of blood in compensation for a transfusion received by a sick relative. Crude seroprevalence did not differ between the two groups.

Supplementary Table 3. Bayesian weighted (unadjusted) and Bayesian weighted, test-performance adjusted seroprevalence estimates for the whole study period (30 April-30 Sept 2020), by sex, age and region.

		Unadjusted seroprevalence		Test-adjusted seroprevalence	
		%	(95% CI)	%	(95% CI)
Sex					
	Male	8.8	8.1-9.6	8.4	7.2-9.5
	Female	7.9	6.7-9.1	7.4	5.9-8.9
Age					
	15 - 24 years	8.0	7.0-9.0	7.5	6.2-8.8
	25 - 34years	8.9	8.0-9.9	8.5	7.2-9.8
	35 - 44 years	8.7	7.8-9.9	8.3	6.9-9.8
	45 - 54 years	7.9	6.4-9.1	7.3	5.5-8.9
	55 - 64 years	7.8	6.1-9.4	7.2	5.2-9.1
Region					
	Central	6.4	4.7-8.4	5.8	3.7-8.0
	Coast	7.7	6.5-9.1	7.2	5.6-8.9
	Eastern / N. Eastern	7.1	5.9-8.5	6.5	4.9-8.2
	Mombasa	13.8	12.1-15.6	13.8	11.7-16.0
	Nairobi	16.4	13.6-19.6	16.7	13.4-20.2
	Nyanza	8.8	7.4-10.3	8.3	6.6-10.2
	Rift Valley	6.6	5.5-7.7	5.9	4.5-7.4
	Western	7.2	4.9-9.9	6.6	3.9-9.7
	National	8.4	7.6-9.1	7.9	6.7-9

Supplementary Table 4. Study periods used in the analysis

Period	Start	Finish	Duration (days)	No. of samples	Median period date
1	30 April 2020	19 June 2020	50	3362	02 June 2020
2	20 June 2020	19 August 2020	60	3837	27 July 2020
3	20 August 2020	30 September 2020	41	3723	01 September 2020

Supplementary note 1: statistical appendix

Bayesian Multi-level Regression with Post-stratification (MRP) was used to account for differences in the age and sex distribution of blood donors and regional differences in the numbers of samples collected over time. This method involves fitting a hierarchical regression model and combining the resulting stratum-specific prevalence estimates with population weights to produce regional and national estimates. Because data on the sensitivity and specificity of the cut-off were also available, we were able to incorporate these parameters and use the model to estimate the "true" prevalence of seropositivity.

Two versions of the hierarchical regression model were fitted: one without a time period effect (Model A) and one with a period effect (Model B). Model A was fitted separately to samples in three periods (30 Apr – 19 Jun, 20 Jun – 19 Aug, 20 Aug – 30 Sept) and Model B was fitted to the combined data.

We simulated 10,000 iterations of 3 chains with a burn in of 1,000 iterations. Convergence was assessed through the R-hat statistic and by visual inspection of the chains.

Model A

$$y_g = \text{Binomial}(n_g, p_g^*)$$

$$p_g^* = se \times p_g + (1 - sp) \times (1 - p_g)$$

$$\text{logit}(p_g) = \beta_{i[g]}^{sex} + \beta_{j[g]}^{region} + \beta_{k[g]}^{age}$$

$$\beta_i^{sex} \sim \text{Normal}(0, 10) \ \text{ for } i = 1, 2$$

$$\beta_j^{region} \sim \text{Normal}(0, \sigma_{region}) \ \text{ for } j = 1, .., 8$$

$$\beta_k^{age} \sim \text{Normal}(0, \sigma_{age}) \text{ for } k = 1, .., 5$$

$$\sigma_{region} \sim \text{Normal}^{+}(0, 0.5)$$

$$\sigma_{age} \sim \text{Normal}^{+}(0, 0.5)$$

$$\text{se} \sim \text{unif}(0, 1)$$

$$sp \sim \text{unif}(0, 1)$$

Region:

1 = central 2 = coast_mombasa 3 = coast_other 4 = eastern_neastern 5 = nairobi 6 = nyanza 7 = rift valley 8 = western

Sex:

1 = female 2 = male

Age group:

1 = 15-24yrs 2 = 25-34yrs 3 = 35-44yrs 4 = 45-54yrs 5 = 55-64yrs

Comments

-The true probability, p_g, of seropositivity in age-sex-region stratum g is related to the observed probability, p_g^* , through the equation $p_g^* = se \times p_g + (1 - sp) \times (1 - p_g)$.

-The effect of sex (β_i^{sex}) is modelled as a fixed effect and the effects of region ($\beta_{j[g]}^{region}$) and age ($\beta_{k[g]}^{age}$) are modelled as random effects. Modelling categorical variables as random effects can improve predictions for categories where only small amounts of data are available [2,3].

- The half-normal priors for σ_{age} and σ_{region} were chosen to be weakly informative [4]. To interpret these priors, note that if the baseline prevalence is 9% then SD = 0.5 on the logit scale corresponds to 95% of estimates being between 3.5% and 21.2%. Non-informative priors were used for all other parameters.

Model B

$$y_{gt} = \text{Binomial}(n_{gt}, p_{gt}^*)$$

$$p_{gt}^* = se \times p_{gt} + (1 - sp) \times (1 - p_{gt})$$

$$\text{logit}(p_{gt}) = \beta_{i[g]}^{sex} + \beta_{j[g]}^{region} + \beta_{k[g]}^{age} + \beta_{j[g],t}^{period}$$

$$\beta_{j,t}^{period} \sim \text{Normal}\big(\gamma_j(t-1), \sigma_{period,j}\big) \text{ for } t = 1,..,10$$

$$\gamma_j \sim \text{Normal}(0, \sigma_{slope})$$

$$\sigma_{period,j} \sim \text{Normal}^+(0, 0.5)$$

$$\sigma_{slope} \sim \text{Normal}^+(0, 0.05)$$

Comments

-The 10 periods correspond approximately to 2-week intervals of time.

-It is assumed that the period effects, $\beta_{j[g],t}^{period}$, follow a region-specific linear trend (γ_j). However, the model allows for some departure from a strict linear trend because the period effects are only constrained to follow a linear trend *on average*.

-The prior for σ_{slope} is equivalent to that of the other SD parameters when the slopes are scaled to reflect change over the whole period.

Model A

```
data {
  int N_se; // denominator sensitivity
  int N_sp; // denominator specificity
  int x; // numerator sensitivity
  int z; // numerator specificity
  int y[5, 2, 8]; // no. seropositives
  int n[5, 2, 8]; // no. samples
  real pw[5, 2, 8]; // proportion of population in each demographic subgroup
  real tot_pw_age[5]; // proportion of population in each age group
  real tot_pw_sex[2]; // proportion female and male
  real tot_pw_region[8]; //proportion of population in each region
}

parameters {
  real<lower=0,upper=1> se;
  real<lower=0,upper=1> sp;
  real bsex[2];
  real bage[5];
  real bregion[8];
  real<lower=0> sd_age;
  real<lower=0> sd_region;
}

transformed parameters {
  real<lower=0,upper=1> p[5, 2, 8];
  real<lower=0,upper=1> p_obs[5, 2, 8];

  for(a in 1:5){
    for(s in 1:2){
      for(r in 1:8){
          p[a, s, r] = inv_logit(bage[a] +
                                 bsex[s] +
                                 bregion[r]);

           p_obs[a, s, r] = se * p[a, s, r] +
                              (1 - sp) * (1 - p[a, s, r]);
         }
       }
     }
}

model {
```

```
  //priors
  se ~ beta(1, 1);
  sp ~ beta(1, 1);
  bsex ~ normal(0, 10);
  bage ~ normal(0, sd_age);
  bregion ~ normal(0, sd_region);
  sd_age ~ normal(0, 0.5);
  sd_region ~ normal(0, 0.5);

 //likelihood

  for(a in 1:5){
   for(s in 1:2){
     for(r in 1:8){
         y[a, s, r] ~ binomial(n[a, s, r], p_obs[a, s, r]);
        }
      }
    }
   x ~ binomial(N_se, se);
   z ~ binomial(N_sp, sp);
}

generated quantities {
real p_national = 0;
vector[5] p_age = rep_vector(0, 5);
vector[2] p_sex = rep_vector(0, 2);
vector[8] p_region = rep_vector(0, 8);

   for(a in 1:5){
     for(s in 1:2){
       for(r in 1:8){
         p_national += p[a, s, r] * pw[a, s, r];
        }
      }
    }

   for(r in 1:8){
     for(a in 1:5){
       for(s in 1:2){
         p_region[r] += p[a, s, r] * pw[a, s, r]/tot_pw_region[r];
        }
      }
    }

   for(a in 1:5){
     for(r in 1:8){
```

```
      for(s in 1:2){
        p_age[a] += p[a, s, r] * pw[a, s, r]/tot_pw_age[a];
        }
      }
    }

  for(s in 1:2){
    for(a in 1:5){
      for(r in 1:8){
        p_sex[s] += p[a, s, r] * pw[a, s, r]/tot_pw_sex[s];
        }
      }
    }

}
```

Model B:

```
data {
  int N_se; //denominator sensitivity
  int N_sp; //denominator specificity
  int x; //numerator sensitivity
  int z; //numerator specificity
  int y[5, 2, 8, 10]; //no. of seropositives
  int n[5, 2, 8, 10]; //no. of samples
  real pw[5, 2, 8]; //proportion of population in each demographic subgroup
  real tot_pw_region[8]; //proportion of population in each region
}

parameters {
  real<lower=0,upper=1> se;
  real<lower=0,upper=1> sp;
  real bsex[2];
  real bage[5];
  real bregion[8];
  real bperiod[8, 10];
  real<lower=0> sd_age;
  real<lower=0> sd_region;
  real<lower=0> sd_period[8];
  vector[8] slope;
}

transformed parameters {
  real<lower=0,upper=1> p[5, 2, 8, 10];
  real<lower=0,upper=1> p_obs[5, 2, 8, 10];
```

```
  for(a in 1:5){
    for(s in 1:2){
      for(r in 1:8){
        for(t in 1:10){
          p[a, s, r, t] = inv_logit(bage[a] +
                                 bsex[s] +
                                 bregion[r] +
                                 bperiod[r, t]);

           p_obs[a, s, r, t] = se * p[a, s, r, t] +
                            (1 - sp) * (1 - p[a, s, r, t]);
          }
        }
      }
    }
}

model {
  //priors
  se ~ beta(1, 1);
  sp ~ beta(1, 1);
  bsex ~ normal(0, 10);
  bage ~ normal(0, sd_age);
  bregion ~ normal(0, sd_region);
  for(r in 1:8){
   for(t in 1:10){
     bperiod[r, t] ~ normal(slope[r] * (t - 1), sd_period[r]);
     }
   }
  sd_age ~ normal(0, 0.5);
  sd_region ~ normal(0, 0.5);
  sd_period ~ normal(0, 0.5);
  slope ~ normal(0, 0.05);

  //likelihood
  for(a in 1:5){
   for(s in 1:2){
    for(r in 1:8){
     for(t in 1:10){
          y[a, s, r, t] ~ binomial(n[a, s, r, t], p_obs[a, s, r, t]);
         }
       }
     }
   }
  x ~ binomial(N_se, se);
  z ~ binomial(N_sp, sp);
```

```
}

generated quantities {
vector[8] exp_slope = exp(slope);
vector[10] p_national = rep_vector(0, 10);
matrix[8, 10] p_region = rep_matrix(0, 8, 10);

  for(t in 1:10){
    for(a in 1:5){
      for(s in 1:2){
        for(r in 1:8){
          p_national[t] += p[a, s, r, t] * pw[a, s, r];
          }
        }
      }
    }

  for(t in 1:10){
    for(r in 1:8){
      for(a in 1:5){
        for(s in 1:2){
          p_region[r, t] += p[a, s, r, t] * pw[a, s, r]/tot_pw_region[r];
          }
        }
      }
    }

}
```

Supplementary References:

1. Uyoga, S., *et al.* Seroprevalence of anti-SARS-CoV-2 IgG antibodies in Kenyan blood donors. *Science* (2020).
2. Gelman, A. & Hill, J. *Data Analysis Using Regression and Multilevel/Hierarchical Models*, (Cambridge University Press, Cambridge, UK, 2007).
3. Gelman, A. & Little, T.C. Poststratification into many categories using hierarchical logistic regression. *Survey Methology* **23**, 217-135 (1997).
4. Gelman, A. Prior distributions for variance parameters in hierarchical models. *Bayesian Analysis* **1**, 515-533 (2006).
