# Supplementary material for: Temporal trends of SARS-CoV-2 seroprevalence during the first wave of the COVID-19 epidemic in Kenya
Source: Nat Commun. 2021 Jun 25;12:3966. doi: 10.1038/s41467-021-24062-3 (PMC8233334; doi:10.1038/s41467-021-24062-3)
Supplement: Supplementary file 2 [file 41467_2021_24062_MOESM3_ESM.pdf]

nature research

Corresponding author(s): Ifedayo Adetifa/Sophie Uyoga

Last updated by author(s): May 12, 2021



# Reporting Summary

Nature Research wishes to improve the reproducibility of the work that we publish. This form provides structure for consistency and transparency in reporting. For further information on Nature Research policies, see our Editorial Policies and the Editorial Policy Checklist.

## Statistics

For all statistical analyses, confirm that the following items are present in the figure legend, table legend, main text, or Methods section.

| n/a | Confirmed | |
|---|---|---|
| ☐ | ☒ | The exact sample size ($n$) for each experimental group/condition, given as a discrete number and unit of measurement |
| ☐ | ☒ | A statement on whether measurements were taken from distinct samples or whether the same sample was measured repeatedly |
| ☐ | ☒ | The statistical test(s) used AND whether they are one- or two-sided<br>*Only common tests should be described solely by name; describe more complex techniques in the Methods section.* |
| ☒ | ☐ | A description of all covariates tested |
| ☐ | ☒ | A description of any assumptions or corrections, such as tests of normality and adjustment for multiple comparisons |
| ☐ | ☒ | A full description of the statistical parameters including central tendency (e.g. means) or other basic estimates (e.g. regression coefficient) AND variation (e.g. standard deviation) or associated estimates of uncertainty (e.g. confidence intervals) |
| ☒ | ☐ | For null hypothesis testing, the test statistic (e.g. $F$, $t$, $r$) with confidence intervals, effect sizes, degrees of freedom and $P$ value noted<br>*Give P values as exact values whenever suitable.* |
| ☐ | ☒ | For Bayesian analysis, information on the choice of priors and Markov chain Monte Carlo settings |
| ☒ | ☐ | For hierarchical and complex designs, identification of the appropriate level for tests and full reporting of outcomes |
| ☒ | ☐ | Estimates of effect sizes (e.g. Cohen's $d$, Pearson's $r$), indicating how they were calculated |

*Our web collection on statistics for biologists contains articles on many of the points above.*

## Software and code

Policy information about availability of computer code

| | |
|---|---|
| Data collection | No software was used |
| Data analysis | Stan Development Team. RStan: the R interface to Stan. R package version 2.23.2 http://mc-stan.org/ |

For manuscripts utilizing custom algorithms or software that are central to the research but not yet described in published literature, software must be made available to editors and reviewers. We strongly encourage code deposition in a community repository (e.g. GitHub). See the Nature Research guidelines for submitting code & software for further information.

## Data

Policy information about availability of data

All manuscripts must include a data availability statement. This statement should provide the following information, where applicable:

- Accession codes, unique identifiers, or web links for publicly available datasets
- A list of figures that have associated raw data
- A description of any restrictions on data availability

The data shown in the manuscript are available upon request from the corresponding author. De-identified data has been published on the Havard dataverse server https://doi.org/10.7910/DVN/FQUNVD





# Field-specific reporting

# Life sciences study design

All studies must disclose on these points even when the disclosure is negative.

| | |
|---|---|
| Sample size | In alignment with the WHO UNITY studies guidance (https://www.who.int/publications/i/item/WHO-2019-nCoV-Seroepidemiology-2020.2), samples size was calculated as follows. Assuming a COVID-19 seroprevalence ranging from 3-10% during the study duration, a sample of 300-500 blood donors per blood transfusion center per month was expected to give a 4-7% margin of error for overall seroprevalence estimates. |
| Data exclusions | Only data from duplicate samples, those from age-ineligible donors and those with missing data were excluded. |
| Replication | Assays on a subset of test samples were repeated at least once on separate days and reproducibility confirmed. Positive and negative control samples are routinely included in all runs and the results from these were reproducible. |
| Randomization | Not applicable to our study which is observational- no intervention here |
| Blinding | Not relevant to our study which is observational- no intervention here |

# Reporting for specific materials, systems and methods

We require information from authors about some types of materials, experimental systems and methods used in many studies. Here, indicate whether each material, system or method listed is relevant to your study. If you are not sure if a list item applies to your research, read the appropriate section before selecting a response.

## Materials & experimental systems

| n/a | Involved in the study | |
|---|---|---|
| ☐ | ☒ | Antibodies |
| ☒ | ☐ | Eukaryotic cell lines |
| ☒ | ☐ | Palaeontology and archaeology |
| ☒ | ☐ | Animals and other organisms |
| ☐ | ☒ | Human research participants |
| ☒ | ☐ | Clinical data |
| ☒ | ☐ | Dual use research of concern |

## Methods

| n/a | Involved in the study | |
|---|---|---|
| ☒ | ☐ | ChIP-seq |
| ☒ | ☐ | Flow cytometry |
| ☒ | ☐ | MRI-based neuroimaging |

## Antibodies

| | |
|---|---|
| Antibodies used | the CR3022 monoclonal antibody (mAb), the anti-SARS-CoV-2 Ab (NIBSC code 20/130), and the convalescent plasma panel NIBSC code 20/118. These were all used at 1:800 dilution, i.e. same dilution as the test samples. CR3022 mAb was produced in-house using plasmids from Krammer (as described in https://science.sciencemag.org/content/sci/suppl/2020/11/10/science.abe1916.DC1/abe1916-Uyoga-SM.pdf). NIBSC 20/130 and NIBSC 20/118 were from National Institute of Biological Standards and Control (NIBSC) UK. |
| Validation | The SARS-CoV-2 antibody assay was developed and validated as reported in full in https://science.sciencemag.org/content/sci/suppl/2020/11/10/science.abe1916.DC1/abe1916-Uyoga-SM.pdf.<br><br>We validated a widely used enzyme-linked immunosorbent assay (ELISA) for SARS-CoV-2 IgG with 910 serum samples from the pre-pandemic period and 174 sera from polymerase chain reaction (PCR)–defined SARS-CoV-2 cases, and a well-characterized five-sera panel from the National Institute of Biological Standards and Control (NIBSC) in the UK. For either receptor-binding domain (RBD) or whole spike, specificity was higher when using a ratio of the sample optical density (OD)/negative control OD than when using the raw sample OD plus 3 standard deviations to define seropositivity. By using OD ratios, both RBD and spike ELISAs correctly classified 901 of 910 prepandemic samples as seronegative. However, the spike ELISA detected more seropositives (166 of 179 compared with 145 of 179 for RBD ELISA) among sera from SARS-CoV-2 PCR-positive individuals. On the basis of these data, we defined anti–SARS CoV-2 IgG seropositivity as an OD ratio >2 and selected the spike ELISA for this study. The sensitivity and specificity, at this threshold, were 92.7% [95% confidence interval (CI), 87.9 to 96.1%] and 99.0% [95% CI, 98.1 to 99.5%], respectively. As previously noted, the RBD and whole-spike ELISA responses were highly correlated, with very little interassay variation. |

# Human research participants

Policy information about studies involving human research participants

| | |
|---|---|
| Population characteristics | The study population is made up of blood donors. The Kenya National Blood Transfusion Services guidelines define eligible blood donors as individuals aged 16-65 years, weighing ≥50kg, with haemoglobin of 12·5g/dl, a normal blood pressure (systolic 120–129 mmHg and diastolic BP of 80–89 mmHg), a pulse rate of 60-100 beats per minute and without any history of illness in the past 6 months. Majority (81%) were males. |
| Recruitment | The study population was recruited as anonymized residual samples from consecutive donor units submitted to the 6 regional centres for transfusion compatibility-testing and infection screening. Since blood donors are restricted to those 16-65 years old, they are not representative of a population sample of all ages which may introduce a selection bias. In addition, to eligibility criteria for blood donation may select for more healthy members of the population which may lead to underestimation of SARS-CoV-2 antibody prevalence. |
| Ethics oversight | The Scientific and Ethics Review Unit of the Kenya Medical Research Institute gave ethical approval |

Note that full information on the approval of the study protocol must also be provided in the manuscript.
